# Highly pathogenic H5N1 avian influenza infection and exposure in waterfowl and waterbirds of the Sacramento National Wildlife Refuge Complex, 2022-2025

**DOI:** 10.3389/fcimb.2026.1993811

**Published:** 2026-10-19

**Authors:** Jeffery D. Sullivan, Rebecca L. Poulson, Michael L. Casazza, Elliott L. Matchett, Michael D’Errico, Alisha Bundy, Jacob Chappell, Desmond Mackell, Andrea Mott, Cory T. Overton, David E. Stallknecht, Diann J. Prosser

**Affiliations:** 1 U.S. Geological Survey, Eastern Ecological Science Center, Laurel, MD, United States; 2 Southeastern Cooperative Wildlife Disease Study, Department of Population Health, College of Veterinary Medicine, The University of Georgia, Athens, GA, United States; 3 U.S. Geological Survey, Western Ecological Research Center, Dixon, CA, United States; 4 U.S. Fish and Wildlife Service, Sacramento National Wildlife Refuge Complex, Willows, CA, United States; 5 California Waterfowl Association, Roseville, CA, United States

**Keywords:** avian influenza, coot, duck, stilt, transmission

## Abstract

The introduction of clade 2.3.4.4b highly pathogenic avian influenza (HPAI) H5N1 into North America in 2021 marked a significant shift in disease dynamics for wild waterfowl populations. To assess infection and seroprevalence trends following this incursion, we leveraged ongoing capture efforts at the Sacramento National Wildlife Refuge Complex between October 2022 and July 2025 to conduct sampling for HPAI H5N1 prevalence and seroprevalence. Our efforts resulted in the analysis of 384 swab and 204 sera samples from assorted waterfowl and waterbird species. HPAI H5N1 infection was confirmed in only three individuals (two Snow Geese and one Greater White-fronted Goose), all asymptomatic at capture. Antibodies to influenza A viruses (IAVs) were detected in all duck and goose species with the majority of positive samples also testing positive for antibodies to both H5 and N1, demonstrating a relatively high population level of previous exposure and likely immunity that aligns with previous findings in these species. This study also reports the first known observation of H5 antibodies in American Coot, though both overall IAV seroprevalence and H5 specific antibodies were notably lower than in sympatric waterfowl. This disparity suggests that American Coot may have lower susceptibility to infection or exhibit behavior that limits exposure compared to sympatric waterfowl. Together these results underscore the value of serologic surveillance as a supportive surveillance strategy to better inform potential management strategies related to HPAI H5N1 risk in wild birds.

## 1 Introduction

The incursion of clade 2.3.4.4b highly pathogenic (HP) H5N1 into North America was followed by rapid and extensive spread of the virus (Harvey et al., 2023), predominately facilitated by wild waterfowl infection and migration (Youk et al., 2023; Jahid and Nolting, 2025). This has resulted in large-scale impacts to wild birds, with over 18,500 individuals from more than 170 species confirmed positive for HP H5N1 infection as of August 2026 (USDA, 2026). Although infections are commonly reported in both ducks and geese, reports of mortality and morbidity have not been consistent even across species. For instance, Mallards (*Anas platyrhynchos*) and other dabbling ducks, the most common reservoirs of low pathogenic (LP) influenza A viruses (IAVs), have been regularly infected with clade 2.3.4.4b HP H5N1, often without displaying clinical symptoms (Spackman et al., 2023; Stallknecht et al., 2024a; Alkie et al., 2025) or apparent impacts on migratory ecology (Teitelbaum et al., 2023). Conversely, there have been large-scale mortality events observed in sea ducks (Avery‐Gomm et al., 2024), diving ducks (Cunningham et al., 2025), and some geese (USDA, 2026; U.S. Geological Survey, 2026). Similarly, avian species that are not traditionally associated with IAV have been widely and variably impacted by the outbreak (Harvey et al., 2023, 2026).

The variation in the reported susceptibility among species is not fully understood but may be related to innate and acquired immunity as well as behavioral attributes that limit exposure. For instance, studies across waterfowl have documented species specific differences in susceptibility as well as infection duration and outcomes (Gonnerman et al., 2024). Acquired immunity related to previous exposure to both LP and HP IAV can result in partial or complete protection to subsequent infection with IAV including HP IAV (Latorre-Margalef et al., 2013; Segovia et al., 2018). The rapid development of population-level antibodies following HP IAV exposure has been demonstrated in many North American dabbling duck, diving duck, and goose species (Stallknecht et al., 2024b, a; Sullivan et al., 2025a, b), though this has been less consistent across non-traditional host species, and data has been lacking (Harvey et al., 2026).

While the U.S. national surveillance strategy provides insight into H5 and H7 viral strains circulating within waterfowl (USDA, 2024), serologic data is required to fully understand disease dynamics within species and populations (Metcalf et al., 2016; Haselbeck et al., 2022). For instance, identifying the prevalence of IAV, H5 and N1 antibodies provides insight into the degree of previous exposure as well as if the population may benefit from homo- or heterosubtypic immunities should there be a subsequent exposure. These data can then help to inform the potential for continued mortality or exposure risk to other susceptible populations (Stallknecht et al., 2024a). Additionally, sampling in non-traditional host species provides insight into the frequency of spillover from waterfowl into these broader avian assemblages not represented in the U.S. national surveillance strategy.

Our objective was to leverage ongoing capture efforts to gain insight into prevalence and seroprevalence of IAV in waterfowl and waterbird species migrating through and wintering on the Sacramento National Wildlife Refuge Complex. Specifically, this study explores HP H5N1 infection and seroprevalence in assorted dabbling ducks and geese as well as coots and stilts captured for multiple ecology studies from October 2022 through July 2025.

## 2 Methods

### 2.1 Study site

Our work was conducted on refuges in the Sacramento National Wildlife Refuge Complex (SNWRC), spanning approximately 70,000 acres of northern California, United States (U.S. Fish and Wildlife Service, 2025). While the complex is comprised of five National Wildlife Refuges (NWRs; Sacramento, Delevan, Colusa, Sutter, and Sacramento River) and three Wildlife Management Areas (Butte Sink, Willow Creek-Lurline, and Steve Thompson North Central Valley), our work was conducted exclusively on the Sacramento (39.43°, -122.18°) and Delevan National Wildlife Refuges (39.31°, -122.10°), which are comprised primarily of managed wetlands (Reiter et al., 2015). Sacramento and Delevan NWRs, along with the broader SNWRC, provide valuable habitat for migratory and wintering waterfowl in the Pacific Flyway. For example, Sacramento NWR alone has historically hosted an estimated 16% of California’s wintering ducks or 10-15% of the Pacific Flyway’s population (Mensik and Paveglio, 2004).

As might be expected given the high density of waterfowl, SNWRC has been subjected to HPAI-related mortality since the incursion of clade 2.3.4.4b HP H5N1. For instance, an event from June 2022 through January 2023 resulted in the death of an estimated 100 Snow Geese (*Anser caerulescens*; WHISPers, 2023), with smaller numbers of mortalities reported across species such as Mallards, Gadwall *(Mareca strepera*), and Canada Geese (*Branta canadensis*) as well as non-waterfowl species such as American White Pelican (*Pelecanus erythrorhynchos*). Similarly, an HPAI outbreak from December 2023 through January 2024 resulted in the death of over 1,100 Snow Geese and 200 Ross’s Geese (*Anser rossii*; WHISPers, 2024). Interestingly, impacts of HPAI infection in wild birds at SNWRC extend beyond mortality, with some Snow Geese failing to migrate or delaying migration following infection (Sullivan et al., 2025b).

### 2.2 Capture and sampling

During July (2025 only), October (2022 – 2024), and December (2022 only) of 2022 through 2025 we captured assorted waterbirds (ducks, geese, coots, and stilts; **Supplementary Table 1**) using rocket nets (Schemnitz et al., 2009) or air boats with dip nets as part of broader movement ecology research (Overton and Casazza, 2023; Casazza et al., 2023). Upon capture, birds were placed into large holding containers and kept out of direct sunlight until they could be processed. Birds were individually banded with U.S. Geological Survey, Bird Banding Laboratory metal bands and data on age and sex were recorded. Some individuals were marked with species appropriate GPS transmitters based upon the specific needs of their related ecological studies.

In addition to banding and marking, one cloacal and one oropharyngeal swab (Ip et al., 2012) were collected from each bird using sterile polyester-tipped applicators and placed as paired samples (by bird in single vial) in 2 mL of cold virus transport medium (VTM, composition previously reported; Poulson and Brown, 2020). Swabs were stored on ice following collection for <72 hours before being shipped to the laboratory where they were held at -80°C until processing. When possible (based on the ecological study’s parameters), serology samples were obtained by taking approximately 0.5-3.0 mL of blood (generally 1.5 mL) from the jugular vein. Blood was placed in a BD Vacutainer SST (BD, Franklin Lakes, New Jersey, USA) and stored on ice (< 12 hours) until centrifuged for 20 minutes at 2500 rpm to separate serum. Serum was shipped to the laboratory where it was held at -20°C until processing.

Appropriate ethical review committee approval was received prior to the capture, sampling, and marking of any birds (USGS EESC IACUC 2022-11P; USGS WERC IACUC Review; and Federal banding permit #21142). All sampling of healthy birds required no use of anesthesia and was conducted as quickly as possible to reduce handling stress. All birds appeared healthy at the time of processing, with no apparent displays of illness or infection typical of waterfowl infected with HPAI (Brown et al., 2006).

### 2.3 Laboratory processing

For HP H5 IAV detection, viral RNA was extracted from swab samples using the MagMAX-96 AI/ND Viral RNA Isolation Kit (Ambion/Applied Biosystems, Foster City, CA) with modifications (Das et al., 2009) and tested using real-time reverse transcriptase-PCR (rRT-PCR) against primers specific for the IAV matrix gene (Spackman, 2020), and/or 2.3.4.4b H5 IAV following previously described methods (Arruda et al., 2024); samples that yielded a cycle threshold (Ct) value <40 in 2.3.4.4b H5 rRT-PCR were submitted to USDA National Veterinary Services Laboratory (NVSL) for official confirmation of subtype and pathogenicity. In addition, some samples were tested by virus isolation in embryonated chicken eggs (Stallknecht et al., 2009; Lebarbenchon et al., 2013) without an initial rRT-PCR screen, though none yielded H5 isolates. HP H5 laboratory testing was completed after captured birds were released; hence, the infection status was not known at time of release. Additional details on the specific assays for each sample are available in our released data (Sullivan et al., 2026).

Serum samples were heat inactivated at 57 °C for 30 minutes and tested using a commercial blocking enzyme-linked immunosorbent assay (bELISA, IDEXX Laboratories, Westbrook, ME) for influenza A virus antibodies as described by the manufacturer. A serum-to-negative control (S/N) absorbance ratio < 0.7 was used as a positive cutoff threshold (Brown et al., 2009; Tolf et al., 2013; Shriner et al., 2016). All sera testing positive on bELISA were further tested for antibodies to H5 by virus microneutralization (MN) and/or hemagglutination inhibition (HI) as described (Stallknecht et al., 2020, 2022). Additionally, in 2022 and 2023 only, sera testing negative via bELISA were also tested for antibodies to H5 by HI and MN (antibody subtyping after these years reserved for only samples meeting S/N threshold to reduce analysis costs based on observation of limited positives beyond threshold – see results). For both HI and MN H5 testing, two reverse genetics (rg) constructs were used as antigens. These included an LP virus with hemagglutinin (HA) and neuraminidase (NA) from A/blue-winged teal/Texas/AI12-4150/2012 (H5N2) and remaining genes from A/Puerto Rico/8/1934 (PR8; H1N1) (this combination of LP H5 on the PR8 backbone is hereafter referred to as BWT). The clade 2.3.4.4b representative antigen [IDCDC-RG71A (H5N8)] consisted of HA and NA from A/Astrakhan/3212/2020 (H5N8) and remaining genes from PR8 (hereafter referred to as AST); the HA contains a modified protease cleavage site characteristic of LP IAV. Sera were also assessed for antibodies to N1 using an enzyme-linked lectin assay (ELLA) as previously described, with A/ruddy turnstone/New Jersey/AI13-2948/2013(H10N1) used as antigen (Stallknecht et al., 2022). Positive threshold titers for HI, MN, and ELLA were 8, 20, and 80, respectively. No statistical comparisons were made due to limited sample sizes.

While all data from Snow Geese published in this manuscript have been previously reported (Sullivan et al., 2025b), they are provided here to allow important comparison across species groups. This is the first reporting for all other data within this manuscript.

## 3 Results

### 3.1 Virus detection

We collected paired oropharyngeal and cloacal swabs from a total of 345 waterfowl and 39 additional waterbirds at the SNWRC from October 2022 – July 2025 (**Table 1**; **Supplementary Table 1**). Only one Greater White-fronted Goose (*Anser albifrons*) and two Snow Geese tested positive for HP H5 RNA. NVSL confirmed these detections as clade 2.3.4.4b HPAI H5N1, however, the Snow Goose samples both failed to meet the Ct < 35 threshold at which NVSL attempts virus isolation (NVSL accession 23-003981). A B3.6 genotype HP H5N1 was isolated from the Greater White-fronted Goose sample (NVSL accession 24-019797). It should be noted that these three birds showed no apparent signs of infection at the time of capture or handling.

**Table 1:** Prevalence (via virus isolation or H5 rRT-PCR) of H5 influenza A virus (IAV) and seroprevalence (via bELISA) of IAV for waterbirds sampled in the Sacramento National Wildlife Refuge Complex from 2022–2025.

			H5 Detections		Antibodies to IAV	
Year	Season	Guild	n	H5+	n	bELISA < 0.7
2022	Fall - Oct	Ducks	65	0	0	–
		Geese	41	0	0	–
	Winter - Dec	Geese	65	2 (3%)	16	14 (88%)
2023	Fall - Oct	Coots	0	–	13	7 (54%)
		Ducks	30	0	32	28 (88%)
		Geese	30	1 (3%)	30	22 (73%)
2024	Fall - Oct	Coots	31	0	21	5 (24%)
		Ducks	100	0	77	35 (45%)
		Stilts	8	0	6	0
2025	Summer - Jul	Ducks	14	0	9	8 (89%)

Duck samples were collected from American Wigeon, Cinnamon Teal, Gadwall, Green-winged Teal, Mallard, Northern Pintail, and Northern Shoveler; goose samples were collected from Greater White-fronted Goose, Ross’s Goose and Snow Goose (species composition is variable across years). Coots and stilts represent American Coot and Black-necked Stilt, respectively.

### 3.2 Serology

We tested 164 individual waterfowl and 40 additional waterbirds, with 107 (65%) and 12 (30%), respectively, testing positive for antibodies to IAV (**Table 1**; **Supplementary Table 1**). For samples collected in October (Fall), when the majority of sampling occurred, IAV antibody prevalence in ducks decreased from 2023 (88%) to 2024 (45%). However, the proportion of IAV antibody positive ducks testing positive for antibodies to both H5 and N1 remained relatively constant from 2023 (64%) to 2024 (80%; **Table 2**). A similar trend in IAV antibody prevalence was observed in American Coots (*Fulica americana*), from 2023 (54%) to 2024 (24%), but prevalence was lower than ducks in both years. Unlike ducks, the proportion of IAV antibody positive coots that tested positive for antibodies to both H5 and N1 was very low in both 2023 (14%) and 2024 (20%). For all species, most of the H5 and N1 antibody positive birds were detected in samples with a bELISA S/N value of <0.7 (**Figure 1**).

**Table 2:** Serological detection of H5 (as detected via hemagglutination inhibition (HI) and virus neutralization (MN), cutoffs ≥8 and ≥20, respectively) and N1 (Enzyme-linked lectin assay, cutoff ≥80) antibodies in wild waterfowl (geese, ducks) and waterbirds (Coots, Stilts) sampled in the Sacramento National Wildlife Refuge Complex from 2022–2025 for birds serum-sample-to-negative-control (S/N) ratios (identified via bELISA) < 0.7.

Year	Season	Guild	n*	HI rg BWT (H5)	HI rg AST (H5)	VN rg BWT (H5)	VN rg AST (H5)	ELLA (N1)	H5^+^	H5 and N1^+^
2022	Winter - Dec	Geese	14	6	12	10	10	12	14 (100%)	12 (86%)
2023	Fall - Oct	Coots	7	0	1	1	3	1	4 (57%)	1 (14%)
		Ducks	28	7	8	15	14	22	23 (82%)	18 (64%)
		Geese	22	4	7	6	5	14	12 (55%)	10 (45%)
2024	Fall - Oct	Coots	5	–	–	1	1	4	1 (20%)	1 (20%)
		Ducks	35	–	–	25	20	30	30 (86%)	28 (80%)
2025	Summer - Jul	Ducks	8	–	–	6	7	8	7 (88%)	7 (88%)

H5 serologic assays were conducted with the clade 2.3.4.4b representative antigen (AST) and an antigen representing North American low pathogenic H5 virus (BWT). Duck samples were collected from American Wigeon, Gadwall, Green-winged Teal, Mallard, Northern Pintail, and Northern Shoveler, while goose samples were collected from Greater White-fronted Goose and Snow Goose (species composition is variable across years). Coots represent American Coot.

^+^ Includes all birds that meet the positive threshold for either AST or BWT. Percentage is reported as # positive divided by the # subtyped. Note that birds positive by one assay may not be positive for another, so cumulative birds positive may be higher than observed in a single assay.

**Figure 1:**
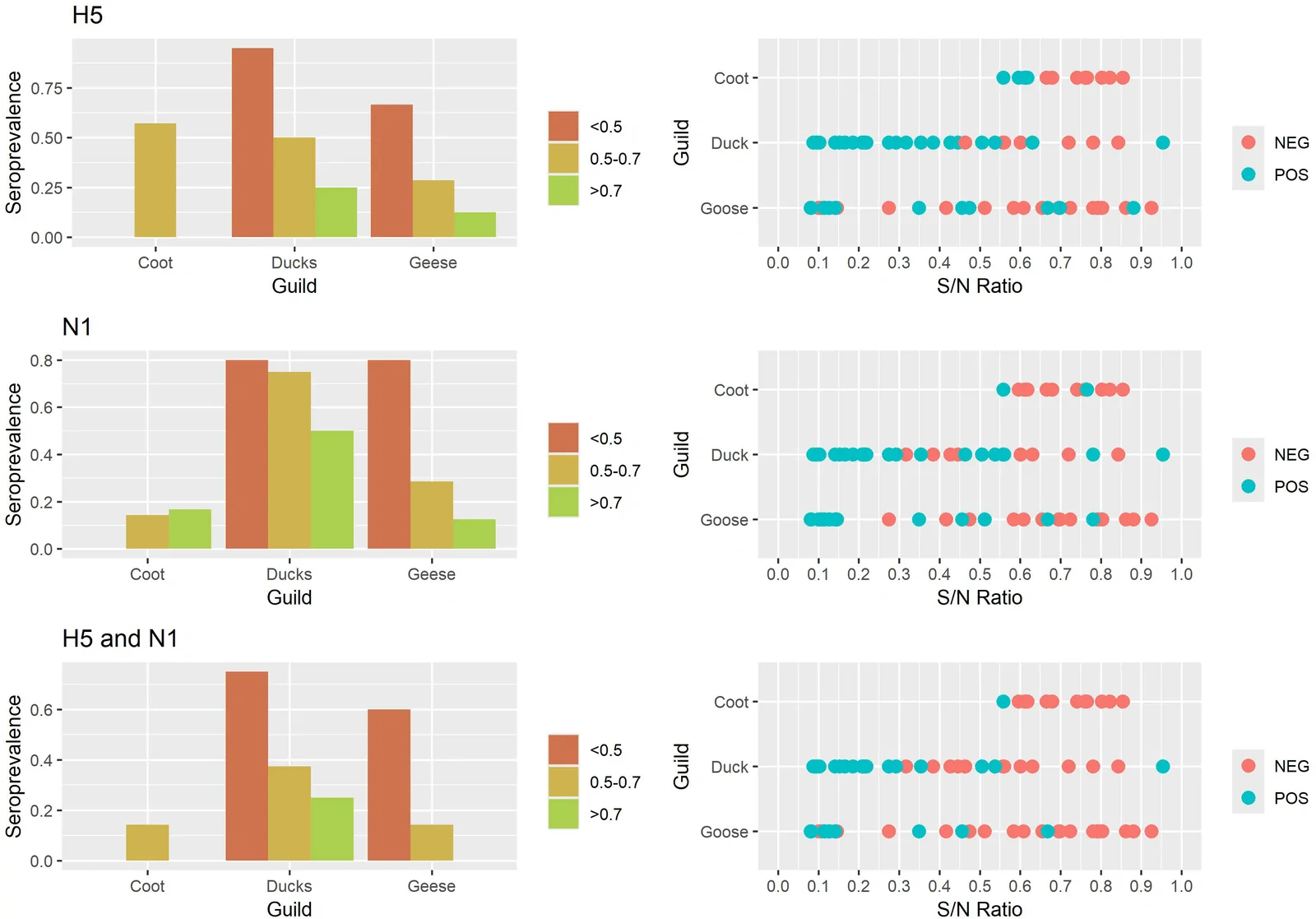
Seroprevalence of H5 (as detected via hemagglutination inhibition (HI) and virus neutralization (MN), cutoffs ≥8 and ≥20, respectively), N1 (Enzyme-linked lectin assay, cutoff ≥80), and both H5 and N1 antibodies as a function of S/N ratio in coots, ducks, and geese sampled in the Sacramento National Wildlife Refuge Complex during October 2023. Duck samples were collected from American Wigeon, Cinnamon Teal, Gadwall, Green-winged Teal, Mallard, Northern Pintail and Northern Shoveler; goose samples were collected from Greater White-fronted Goose, Ross’s Goose and Snow Goose (species composition is variable across years). Coots represent American Coot.

## 4 Discussion

Our data support previous findings that, following the incursion of clade 2.3.4.4b HP H5N1, a large proportion of ducks, much like Snow Geese at this refuge (Sullivan et al., 2025b), have detectable antibodies to both H5 and N1 indicating a potentially high rate of exposure to HP H5N1 and subsequent survival (Stallknecht et al., 2024a, 2026; Sullivan et al., 2025a). This observation is not unique to this refuge or the Pacific Flyway and has been observed with numerous duck species in the Mississippi Flyway where antibodies to H5 and N1 increased dramatically following the incursion of 2.3.4.4b HP H5N1 (Stallknecht et al., 2024b, a). It is not possible with the serologic techniques used in this study to know if these H5 or N1 antibodies are solely the result of exposure to HP H5N1, and it is likely that they represent a history of exposure to both HP H5, LP H5, and with N1 (Vatti et al., 2017). It has previously been reported that LP H5 IAV are commonly detected in these wetlands and it has been suggested that LP H5 IAV could be maintained locally via non-migratory waterfowl (Hill et al., 2012).

The low prevalence of antibodies to IAV in coots has been previously reported (De Marco et al., 2005). Indeed, this may be the first identification of H5 antibodies in this species. There are also few reports of coot mortality related to HP H5N1 (USDA, 2026). However, Coot are not part of the U.S. national active surveillance strategy (USDA, 2025) and therefore detections rely on passive surveillance, of which the remote ecological nature of this species may reduce detections if they are to occur. The difference in antibody prevalence between waterfowl and coots occupying the same habitat cannot be explained directly by our data but may relate to differences in innate susceptibility or behavior traits affecting exposure (De Marco et al., 2005). While it is possible that the low detected seroprevalence is a result of survivorship bias (i.e., the majority exposed individuals died resulting in low population level antibodies), we feel this is unlikely given the low number of American Coot mortalities observed during the course of this outbreak (USDA, 2026).

Our data align with previous studies (Brown et al., 2009; Tolf et al., 2013; Shriner et al., 2016) and support the use of an S/N < 0.7 threshold for interpreting bELISA results and guiding further serotyping assays. While some individuals with S/N > 0.7 tested positive for H5 and N1 antibodies, this was only a small percentage (6% or 1 of 18; though samples with S/N > 0.7 were not subtyped in all years) of the samples. It is important to recognize that we cannot know how long before a sampling event an individual was infected with an IAV and it is possible that test sensitivity will vary over time and among different testing formats. As such, a S/N < 0.7 threshold is not a perfect screening tool but should not result in significant error related to detecting subtype specific antibodies.

Translating the significance of antibodies to IAV, HP H5, LP H5, and N1 to population immunity is complicated, but these data do provide insight into possible outcomes related to subsequent HP H5N1 infection. Based on experimental studies involving both LP and HP IAV sequential challenges, previous exposures can result in reduced infection, mortality, and viral shedding during subsequent infections with both LP and HP H5 IAV (Kalthoff et al., 2008; Fereidouni et al., 2009; Berhane et al., 2014; Latorre-Margalef et al., 2017; Tarasiuk et al., 2023). Antibodies to both H5 and N1 were detected in 64% and 80% of ducks sampled in 2023 and 2024, respectively. This relatively high level of previous subtype specific immunity may partially explain the lack of detected HP H5N1 infection during both years. Interestingly, a high proportion of ducks (88%) sampled during July 2025 also had antibodies to both H5 and N1. This observation is similar to results from Snow geese sampled at this site that failed to migrate during the summer of 2023 where 100% had antibodies to H5 (Sullivan et al., 2025b). In contrast to ducks, antibodies to IAV, H5, and N1 were rarely detected in both American Coots and Black-necked Stilts (*Himantopus mexicanus*) despite their occupying the same aquatic habitats. As identified above, this may indicate a low probability of HP H5N1 mortality in these two species as they are either unlikely to be exposed or there is some process limiting infection. Still, based on antibody data, both species were essentially naïve and as such do remain susceptible to infection if the lack of antibodies is in some way driven by a lack of exposure to the virus, even if this appears unlikely given the degree of circulation in sympatric waterfowl (though data are stronger for coot than stilts, for which sampling was especially limited).

Because there was not a clear antigenic difference observed in both HI and MN results using the representative LP and HP H5 antigens, we cannot definitively estimate specific exposure to LP and HP H5. It is possible that potential antigenic differences were masked by cross reactive antibodies resulting from previous exposure to multiple IAV subtypes as well as antigenic imprinting (Vatti et al., 2017). An additional limitation to our data is a limited sample size collected from a restricted period of the year, predominately within a single month per species, which can provide a limited view of viral prevalence, seroprevalence, and impacts.

Overall, these serologic data indicate a high level of IAV immunity in waterfowl that, based on experimental trials, should reduce the potential risk for widespread or seasonally related mortality. Results from coots and stilts also highlight the species differences that exist related to exposure and potential risk at a given site and time. While additional sampling is required to understand how these data change in subsequent seasons, they provide insight into the baseline immunological responses in the years following the incursion of clade 2.3.4.4b HP H5N1.

## Data Availability

The datasets generated for this study can be found in a USGS Data Release (https://doi.org/10.5066/P1FWYEB8).

## References

[B1] Alkie T. N. Embury-Hyatt C. Signore A. V. Baldwin F. Hisanaga T. Xu W. . (2025). Comparative pathogenicity of three A(H5N1) clade 2.3.4.4b HPAI viruses in blue-winged teal and transmission to domestic poultry. Am. Soc. For. Microbiol. mSphere. 10. doi: 10.1128/msphere.00021-25 40401902 PMC12188715

[B2] Arruda B. Vincent Baker A. L. Buckley A. Anderson T. K. Torchetti M. Bergeson N. H. . (2024). Divergent pathogenesis and transmission of highly pathogenic avian influenza A(H5N1) in swine. Centers. For. Dis. Control. Prev. (CDC). Emerg. Infect. Dis. 30, 738–751. doi: 10.3201/eid3004.231141 38478379 PMC10977838

[B3] Avery-Gomm S. Barychka T. English M. Ronconi R. A. Wilhelm S. I. Rail J. . (2024). Wild bird mass mortalities in eastern Canada associated with the highly pathogenic avian influenza A(H5N1) virus 2022. Ecosphere 15. doi: 10.1002/ecs2.4980

[B4] Berhane Y. Embury-Hyatt C. Leith M. Kehler H. Suderman M. Pasick J. (2014). Pre-exposing Canada Geese (Branta canadensis) to a low-pathogenic H1N1 avian influenza virus protects them against H5N1 HPAI virus challenge. J. Wildl. Dis. 50, 84–97. doi: 10.7589/2012-09-237 24171557

[B5] Brown J. D. Stallknecht D. E. Beck J. R. Suarez D. L. Swayne D. E. (2006). Susceptibility of North American ducks and gulls to H5N1 highly pathogenic avian influenza viruses. Emerg. Infect. Dis. 12, 1663–1670. doi: 10.3201/eid1211.060652 PMC3372354 17283615

[B6] Brown J. D. Stallknecht D. E. Berghaus R. D. Luttrell M. P. Velek K. Kistler W. . (2009). Evaluation of a commercial blocking enzyme-linked immunosorbent assay to detect avian influenza virus antibodies in multiple experimentally infected avian species. Clin. Vaccine Immunol. 16, 824–829. doi: 10.1128/cvi.00084-09 19386796 PMC2691043

[B7] Casazza M. L. Lorenz A. A. Overton C. T. Matchett E. L. Mott A. L. Mackell D. A. . (2023). AIMS for wildlife: Developing an automated interactive monitoring system to integrate real-time movement and environmental data for true adaptive management. Acad. Press J. Environ. Manage. 345. doi: 10.1016/j.jenvman.2023.118636 37574637

[B8] Cunningham M. W. Brown J. Hardman R. Loerzel S. Kluever B. M. Zachariah T. T. . (2025). Outbreaks of highly pathogenic H5N1 influenza A virus infection in Black Vultures (Coragyps atratus), USA 2022. Wildlife. Dis. Ass. Inc. J. Wildl. Dis. 61, 955–966. doi: 10.7589/jwd-d-24-00214 40639804

[B9] Das A. Spackman E. Pantin-Jackwood M. J. Suarez D. L. (2009). Removal of real-time reverse transcription polymerase chain reaction (RT-PCR) inhibitors associated with cloacal swab samples and tissues for improved diagnosis of avian influenza virus by RT-PCR. J. Vet. Diagn. Investiagtion., 771–778. doi: 10.1177/104063870902100603 19901277

[B10] De Marco M. A. Foni E. Campitelli L. Delogu M. Raffini E. Chiapponi C. . (2005). Influenza virus circulation in wild aquatic birds in Italy during H5N2 and H7N1 poultry epidemic periods, (1998 to 2000). Avian Pathol. 34, 480–485. doi: 10.1080/03079450500368185 16537162

[B11] Fereidouni S. R. Starick E. Beer M. Wilking H. Kalthoff D. Grund C. . (2009). Highly pathogenic avian influenza virus infection of mallards with homo- and heterosubtypic immunity induced by low pathogenic avian influenza viruses. http://www.pubmedcentral.nih.gov/articlerender.fcgi?artid=2724736&tool=pmcentrez&rendertype=abstract (Accessed March 2013). 10.1371/journal.pone.0006706 PMC2724736 19693268

[B12] Gonnerman M. Leyson C. Sullivan J. D. Pantin-Jackwood M. J. Spackman E. Mullinax J. M. . (2024). A systematic review of laboratory investigations into the pathogenesis of avian influenza viruses in wild avifauna of North America. Proc. R. Soc. B. Biol. Sci. 291. doi: 10.1098/rspb.2024.1845 PMC11521597 39471857

[B13] Harvey J. A. Gonnerman M. Yin S. Kent C. M. Cullen J. Sullivan J. D. . (2026). Feathers and flu: Identifying data gaps in avian influenza host dynamics to prioritize wildlife conservation. Wildl. Monogr. doi: 10.1002/wmon.7001542587375

[B14] Harvey J. A. Mullinax J. M. Runge M. C. Prosser D. J. (2023). The changing dynamics of highly pathogenic avian influenza H5N1: Next steps for management & science in North America. Biol. Conserv. 282, 110041. doi: 10.1016/j.biocon.2023.110041

[B15] Haselbeck A. H. Im J. Prifti K. Marks F. Holm M. Zellweger R. M. (2022). Serology as a tool to assess infectious disease landscapes and guide public health policy. MDPI. doi: 10.3390/pathogens11070732 35889978 PMC9323579

[B16] Hill N. J. Takekawa J. Y. Ackerman J. T. Hobson K. A. Herring G. Cardona C. J. . (2012). Migration strategy affects avian influenza dynamics in mallards (Anas platyrhynchos). http://www.ncbi.nlm.nih.gov/pubmed/22971007 (Accessed March 2013). 10.1111/j.1365-294X.2012.05735.x 22971007

[B17] Ip H. S. Dusek R. J. Heisey D. M. (2012). The effect of swab sample choice on the detection of avian influenza in apparently healthy wild ducks. Avian Dis. 56, 114–119. doi: 10.1637/10011-983211-digest.1 22545536

[B18] Jahid M. J. Nolting J. M. (2025). Dynamics of a panzootic: Genomic insights, host range, and epidemiology of the highly pathogenic avian influenza A(H5N1) clade 2.3.4.4b in the United States. Viruses 17, 312. doi: 10.3390/v17030312 PMC11946527 40143242

[B19] Kalthoff D. Breithaupt A. Teifke J. P. Globig A. Harder T. Mettenleiter T. C. . (2008). Highly pathogenic avian influenza virus (H5N1) in experimentally infected adult mute swans. Emerg. Infect. Dis. 10.3201/eid1408.080078 PMC2600380 18680652

[B20] Latorre-Margalef N. Brown J. D. Fojtik A. Poulson R. L. Carter D. França M. S. . (2017). Competition between influenza A virus subtypes through heterosubtypic immunity modulates re-infection and antibody dynamics in the mallard duck. PloS Pathog. doi: 10.1371/journal.ppat.1006419 PMC5481145 28640898

[B21] Latorre-Margalef N. Grosbois V. Wahlgren J. Munster V. J. Tolf C. Fouchier R. A. M. . (2013). Heterosubtypic immunity to influenza A virus infections in mallards may explain existence of multiple virus subtypes. PloS Pathog. doi: 10.1371/journal.ppat.1003443 23818849 PMC3688562

[B22] Lebarbenchon C. Poulson R. Shannon K. Slagter J. Slusher M. J. Wilcox B. R. . (2013). Isolation of influenza A viruses from wild ducks and feathers in Minnesota, (2010 – 2011). Avian Dis. 57, 677–680. doi: 10.1637/10455-112512-resnote.1 24283137 PMC11327954

[B23] Mensik J. G. Paveglio F. L. (2004). Biological integrity, diversity, and environmental health policy and the attainment of refuge purposes: A Sacramento National Wildlife Refuge case study. Nat. Resour. J. 44. Available online at: https://digitalrepository.unm.edu/nrj/vol44/iss4/12.

[B24] Metcalf C. J. E. Farrar J. Cutts F. T. Basta N. E. Graham A. L. Lessler J. . (2016). Use of serological surveys to generate key insights into the changing global landscape of infectious disease. Lancet Publishing. Group. doi: 10.1016/s0140-6736(16)30164-7 27059886 PMC5678936

[B25] Overton C. T. Casazza M. L. (2023). Movement behavior, habitat selection, and functional responses to habitat availability among four species of wintering waterfowl in California. Front. Media. SA. Front. Ecol. Evol. 11. doi: 10.3389/fevo.2023.1232704

[B26] Poulson R. L. Brown J. D. (2020). “ Wild bird surveillance for avian influenza virus, 3rd edition,” in Animal Influenza Virus: Methods and Protocols. Ed. Spackman E. (Hatfield, Hertfordshire: Humana Press), 93–112. 10.1007/978-1-0716-0346-8_8 32170683

[B27] Reiter M. E. Wolder M. A. Isola J. E. Jongsomjit D. Hickey C. M. Carpenter M. . (2015). Local and landscape habitat associations of shorebirds in wetlands of the Sacramento Valley of California. U.S. Fish. Wildlife. Service. J. Fish. Wildl. Manag. 6, 29–43. doi: 10.3996/012014-jfwm-003

[B28] Schemnitz S. D. Batcheller G. R. Lovallo M. J. White H. B. Fall M. W. (2009). “ Capturing and handling wild animals,” in The Wildlife Techniques Manual. Ed. Silvy N. J. ( John Hopkins University Press, Baltimore), 232–269.

[B29] Segovia K. M. França M. S. Leyson C. L. Kapczynski D. R. Chrzastek K. Bahnson C. S. . (2018). Heterosubtypic immunity increases infectious dose required to infect mallard ducks with influenza A virus. PloS One 20. doi: 10.1371/journal.pone.0196394 29698449 PMC5919434

[B30] Shriner S. A. VanDalen K. K. Root J. J. Sullivan H. J. (2016). Evaluation and optimization of a commercial blocking ELISA for detecting antibodies to influenza A virus for research and surveillance of mallards. Elsevier. B.V. J. Virol. Methods 228, 130–134. doi: 10.1016/j.jviromet.2015.11.021 26645952

[B31] Spackman E. (2020). “ Avian influenza virus detection and quantitation by real-time RT-PCR,” in Animal Influenza Virus: Methods and Protocols, 137–148. 10.1007/978-1-0716-0346-8_11 32170686

[B32] Spackman E. Pantin-Jackwood M. J. Lee S. A. Prosser D. (2023). The pathogenesis of a 2022 North American highly pathogenic clade 2.3.4.4b H5N1 avian influenza virus in mallards (Anas platyrhynchos). Avian Pathol., 1–10. Available online at: https://www.tandfonline.com/doi/full/10.1080/03079457.2023.2196258. 10.1080/03079457.2023.2196258 36999798

[B33] Stallknecht D. E. Carter D. L. Blake-Bradshaw A. G. Masto N. M. Highway C. J. Feddersen J. C. . (2024a). Influenza A virus antibodies in ducks and introduction of highly pathogenic influenza A(H5N1) virus, Tennessee, USA. Centers. For. Dis. Control. Prev. (CDC). Emerg. Infect. Dis. 30, 2647–2650. doi: 10.3201/eid3012.241126 PMC11616651 39592421

[B34] Stallknecht D. E. Carter D. L. Sullivan-Brügger L. Cao Z. Link P. Rome K. . (2026). Seasonal infection and antibody response to highly pathogenic H5N1 influenza A virus in ducks in the Mississippi and Central Flyways, United States. Can. Sci. Publishing. Can. J. Microbiol. 72, 1–10. doi: 10.1139/cjm-2025-0274 41941827

[B35] Stallknecht D. E. Carter D. L. Sullivan-Brügger L. Link P. Ferraro E. McCarty C. . (2024b). Highly pathogenic H5N1 influenza A virus (IAV) in blue-winged teal in the Mississippi Flyway is following the historic seasonal pattern of low-pathogenicity IAV in ducks. Multidiscip. Digital. Publishing. Ins. (MDPI). Pathog. 13. doi: 10.3390/pathogens13111017 PMC11597780 39599570

[B36] Stallknecht D. E. Fojtik A. Carter D. L. Crum-Bradley J. A. Perez D. R. Poulson R. L. (2022). Naturally acquired antibodies to influenza A virus in fall-migrating North American mallards. MDPI. Vet. Sci. 9. doi: 10.3390/vetsci9050214 35622742 PMC9148056

[B37] Stallknecht D. E. Kienzle-Dean C. Davis-Fields N. Jennelle C. S. Bowman A. S. Nolting J. M. . (2020). Limited detection of antibodies to clade 2.3.4.4 A/Goose/Guangdong/1/1996 lineage highly pathogenic H5 avian influenza virus in NorthAmerican waterfowl. J. Wildl. Dis. 56, 47–57. doi: 10.7589/2019-01-003 31556839 PMC8761256

[B38] Stallknecht A. D. E. Shane S. M. Zwank P. J. Senne D. A. Kearney M. T. (2009). Avian influenza viruses from migratory and resident ducks of coastal Louisiana. Avian Dis. 34, 398–405. doi: 10.2307/1591427 2369380

[B39] Sullivan J. D. Casazza M. L. Poulson R. L. Matchett E. L. Overton C. T. Carpenter M. . (2025b). Potential impacts of 2.3.4.4b highly pathogenic H5N1 avian influenza virus infection on Snow Goose (Anser caerulescens) movement ecology. PloS One 20, e0328149. Available online at: https://dx.plos.org/10.1371/journal.pone.0328149. 10.1371/journal.pone.0328149 PMC12303340 40720487

[B40] Sullivan J. D. Poulson R. L. Casazza M. L. Matchett E. L. D'Errico M. Bundy A. . (2026). Data describing H5N1 avian influenza infection and exposure in waterfowl and waterbirds of the Sacramento National Wildlife Refuge Complex: U.S. Geological Survey data release. doi: 10.5066/P1FWYEB8

[B41] Sullivan J. Poulson R. Olsen G. H. Berlin A. M. Cao Z. Carter D. L. . (2025a). Rapid increase in antibodies to influenza A virus H5 and N1 in lesser scaup (Aythya affinis) following the introduction of 2.3.4.4b H5N1 into North America. Can. Sci. Publishing. Can. J. Microbiol. doi: 10.1139/cjm-2025-0176 41232085

[B42] Tarasiuk K. Kycko A. Świętoń E. Bocian Ł. Wyrostek K. Śmietanka K. (2023). Homo- and heterosubtypic immunity to low pathogenic avian influenza virus mitigates the clinical outcome of infection with highly pathogenic avian influenza H5N8 clade 2.3.4.4.b in captive mallards (Anas platyrhynchos). Pathogens 12, 217. Available online at: https://www.mdpi.com/2076-0817/12/2/217. 10.3390/pathogens12020217 PMC9964785 36839489

[B43] Teitelbaum C. S. Masto N. M. Sullivan J. D. Keever A. C. Poulson R. L. Carter D. L. . (2023). North American wintering mallards infected with highly pathogenic avian influenza show few signs of altered local or migratory movements. Sci. Rep. 13, 14473. Available online at: https://www.nature.com/articles/s41598-023-40921-z. 10.1038/s41598-023-40921-z PMC10475108 37660131

[B44] Tolf C. Latorre-Margalef N. Wille M. Bengtsson D. Gunnarsson G. Grosbois V. . (2013). Individual variation in influenza A virus infection histories and long-term immune responses in mallards. PloS One 8. doi: 10.1371/journal.pone.0061201 23637797 PMC3634042

[B45] USDA (2024). Implementation Plan for Avian Influenza Surveillance in Waterfowl in the United States.

[B46] USDA (2025). Implementation Plan for Avian Influenza Surveillance in Waterfowl in the United States: Summer 2025 - Spring 2026.

[B47] USDA (2026). 2022-2026 Detections of Highly Pathogenic Avian Influenza in Wild Birds.

[B48] US Fish and Wildlife Service (2025). Sacramento National Wildlife Refuge.

[B49] US Geological Survey (2026). Wildlife Health Information Sharing Partnership: Event Reporting System.

[B50] Vatti A. Monsalve D. M. Pacheco Y. Chang C. Anaya J. M. Gershwin M. E. (2017). Original antigenic sin: A comprehensive review. Acad. Press. doi: 10.1016/j.jaut.2017.04.008 28479213

[B51] WHISPers (2023). Event 202438.

[B52] WHISPers (2024). Event 203680.

[B53] Youk S. Torchetti M. K. Lantz K. Lenoch J. B. Killian M. L. Leyson C. . (2023). H5N1 highly pathogenic avian influenza clade 2.3.4.4b in wild and domestic birds: Introductions into the United States and reassortments, December 2021–April 2022. Virology 587, 109860. Available online at: https://linkinghub.elsevier.com/retrieve/pii/S0042682223001733. 10.1016/j.virol.2023.109860 37572517

