# Supplementary material for: Highly pathogenic H5N1 avian influenza infection and exposure in waterfowl and waterbirds of the Sacramento National Wildlife Refuge Complex, 2022-2025
Source: Front Cell Infect Microbiol. 2026 Oct 19;16:1993811. doi: 10.3389/fcimb.2026.1993811 (PMC13724457; doi:10.3389/fcimb.2026.1993811)
Supplement: Supplementary file 1 [file Table1.docx]

Supplementary Material

# for

Highly Pathogenic H5N1 Avian Influenza Infection and Exposure in Waterfowl and Waterbirds of the Sacramento National Wildlife Refuge Complex, 2022-2025.

Jeffery D. Sullivan^1^, Rebecca L. Poulson^2^, Michael L. Casazza^3^, Elliott L. Matchett^3^, Michael D’Errico^4^, Alisha Bundy^3^, Jacob Chappell^3^, Desmond Mackell^3^, Andrea Mott^3^, Cory T. Overton^3^, David E. Stallknecht^2,*^, Diann J. Prosser^1,*^

^1^U.S. Geological Survey, Eastern Ecological Science Center, Laurel, Maryland, USA

^2^Southeastern Cooperative Wildlife Disease Study, College of Veterinary Medicine, Department of Population Health, The University of Georgia, Athens, Georgia, USA

^3^U.S. Geological Survey, Western Ecological Research Center, Dixon, California, USA

^4^U.S. Fish and Wildlife Service, Sacramento National Wildlife Refuge Complex, Willows, California, USA

The use of trade, product, or firm names in this publication is for descriptive purposes only and does not imply endorsement by the U.S. Government.

# Supplementary Table 1. Prevalence (via virus isolation or H5 rRT-PCR) of H5 influenza A virus (IAV) and seroprevalence (via bELISA) of IAV for waterbirds sampled in the Sacramento National Wildlife Refuge Complex from 2022–2025. Duck samples were collected from American Wigeon (AMWI), Cinnamon Teal (CITE), Gadwall (GADW), Green-winged Teal (AGWT), Mallard (MALL), Northern Pintail (NOPI), and Northern Shoveler (NOSH); goose samples were collected from Greater White-fronted Goose (GWFG), Ross’s Goose (ROGO) and Snow Goose (SNGO). Broader waterbirds include American Coot (AMCO) and Black-necked Stilt (BNST), respectively.

|  |  |  |  |  | H5 Detections | |  | Antibodies | |
| --- | --- | --- | --- | --- | --- | --- | --- | --- | --- |
| Year | Season | Species | Age |  | n | H5+ |  | n | bELISA < 0.7 |
| 2022 | Fall - Oct | GWFG | AHY |  | 35 | 0 |  | 0 | - |
| 2022 | Fall - Oct | MALL | AHY |  | 20 | 0 |  | 0 | - |
| 2022 | Fall - Oct | MALL | HY |  | 1 | 0 |  | 0 | - |
| 2022 | Fall - Oct | NOPI | AHY |  | 44 | 0 |  | 0 | - |
| 2022 | Fall - Oct | SNGO | AHY |  | 6 | 0 |  | 0 | - |
| 2022 | Winter - Dec | GWFG | AHY |  | 5 | 0 |  | 0 | - |
| 2022 | Winter - Dec | ROGO | AHY |  | 10 | 0 |  | 0 | - |
| 2022 | Winter - Dec | SNGO | AHY |  | 44 | 1 |  | 10 | 10 |
| 2022 | Winter - Dec | SNGO | HY |  | 6 | 1 |  | 6 | 4 |
| 2023 | Fall - Oct | AMCO | AHY |  | 0 | - |  | 11 | 7 |
| 2023 | Fall - Oct | AMCO | HY |  | 0 | - |  | 2 | 0 |
| 2023 | Fall - Oct | AMWI | AHY |  | 0 | - |  | 2 | 2 |
| 2023 | Fall - Oct | GWFG | AHY |  | 30 | 1 |  | 30 | 22 |
| 2023 | Fall - Oct | NOPI | AHY |  | 30 | 0 |  | 30 | 26 |
| 2024 | Fall - Oct | AMCO | AHY |  | 31 | 0 |  | 21 | 5 |
| 2024 | Fall - Oct | AGWT | AHY |  | 14 | 0 |  | 10 | 5 |
| 2024 | Fall - Oct | AMWI | AHY |  | 6 | 0 |  | 5 | 4 |
| 2024 | Fall - Oct | AMWI | HY |  | 2 | 0 |  | 1 | 1 |
| 2024 | Fall - Oct | BNST | AHY |  | 2 | 0 |  | 1 | 0 |
| 2024 | Fall - Oct | BNST | HY |  | 6 | 0 |  | 5 | 0 |
| 2024 | Fall - Oct | CITE | AHY |  | 1 | 0 |  | 0 | - |
| 2024 | Fall - Oct | GADW | AHY |  | 16 | 0 |  | 13 | 2 |
| 2024 | Fall - Oct | GADW | HY |  | 10 | 0 |  | 8 | 0 |
| 2024 | Fall - Oct | MALL | AHY |  | 7 | 0 |  | 4 | 2 |
| 2024 | Fall - Oct | NOPI | AHY |  | 12 | 0 |  | 10 | 6 |
| 2024 | Fall - Oct | NOSH | AHY |  | 29 | 0 |  | 23 | 15 |
| 2024 | Fall - Oct | NOSH | HY |  | 3 | 0 |  | 3 | 0 |
| 2025 | Summer - Jul | CITE | AHY |  | 3 | 0 |  | 0 | - |
| 2025 | Summer - Jul | MALL | AHY |  | 11 | 0 |  | 9 | 8 |
